# No evidence of spread of Linda pestivirus in the wild boar population in Southern Germany

**DOI:** 10.1186/s12985-024-02476-x

**Published:** 2024-08-30

**Authors:** Doreen Schulz, Andrea Aebischer, Kerstin Wernike, Martin Beer

**Affiliations:** Friedrich-Loeffler-Institut, Südufer 10, 17493 Greifswald-Insel Riems, Germany

**Keywords:** Linda virus, Pestivirus, *Suidae*, Epidemiology, Serology, Reverse genetics, Chimeric virus

## Abstract

Lateral-shaking inducing neuro-degenerative agent virus (LindaV) is a novel member of the highly diverse genus *Pestivirus* within the family *Flaviviridae*. LindaV was first detected in Austria in 2015 and was associated with congenital tremor in piglets. Since then, the virus or specific antibodies have been found in a few further pig farms in Austria. However, the actual spatial distribution and the existence of reservoir hosts is largely unknown. Since other pestiviruses of pigs such as classical swine fever virus or atypical porcine pestivirus can also infect wild boar, the question arises whether LindaV is likewise present in the wild boar population. Therefore, we investigated the presence of neutralizing antibodies against LindaV in 200 wild boar samples collected in Southern Germany, which borders Austria. To establish a serological test system, we made use of the interchangeability of the surface glycoproteins and created a chimeric pestivirus using Bungowannah virus (species *Pestivirus australiaense*) as synthetic backbone. The E1 and E2 glycoproteins were replaced by the heterologous E1 and E2 of LindaV resulting in the chimera BV_E1E2_LV. Viable virus could be rescued and was subsequently applied in a neutralization test. A specific positive control serum generated against the E2 protein of LindaV gave a strong positive result, thereby confirming the functionality of the test system. All wild boar samples, however, tested negative. Hence, there is no evidence that LindaV has become highly prevalent in the wild boar population in Southern Germany.

## Introduction

The genus *Pestivirus* of the family *Flaviviridae* comprises highly variable RNA viruses with a great socio-economic significance for livestock farming globally. The four classical pestivirus species, namely bovine viral diarrhea virus (BVDV) types 1 and 2 (species *Pestivirus bovis* and *Pestivirus tauri*), classical swine fever virus (CSFV, species *Pestivirus suis*), and border disease virus (BDV, species *Pestivirus ovis*) were initially described in the 19th or in the middle of the 20th century [1–3] and infect cloven-hoofed animals. During the last two decades, however, the phylogenetic and host diversity of pestiviruses expanded considerably, as novel, highly diverse members of this genus were found in a wide range of animals from antelopes to rats or even bats and whales [4–7]. In addition, in swine, pestiviruses have been identified that are genetically and antigenically distinct from the four classical pestivirus species. In 2015, the atypical porcine pestivirus (APPeV, species *Pestivirus scrofae*) was described in North America [8] and has since been reported in multiple countries on the American, European and Asian continent [9]. APPeV has been associated with a variety of clinical presentations, including congenital tremor (CT) in piglets and reproductive disorders, but was also found in clinically inconspicuous pigs. Two other newly discovered atypical pestiviruses of pigs are Bungowannah virus (BuPV, species *Pestivirus australiaense*) and lateral-shaking inducing neuro-degenerative agent virus (LindaV, species *Pestivirus L*) [10, 11]. Bungowannah virus may cause cardiac failure, stillbirth, and sudden death in piglets [12] and LindaV has been associated with CT in piglets [11]. While APPeV occurs worldwide [9], BuPV and LindaV appear to be restricted to individual farms in Australia and Austria, respectively [10, 11, 13–17]. The emergence of these atypical pestiviruses highlights the importance of continuous surveillance and monitoring of viral pathogens in swine populations. Improved diagnostic tools and comprehensive studies of the molecular biology and epidemiology of these viruses and the identification of potential reservoir hosts are necessary to better understand the viruses’ impact on pig health and production.

Natural hosts of the classical pestivirus CSFV are all members of the family *Suidae* and wild boar populations have been identified as a significant source of virus transmission to domestic pigs [18, 19]. APPeV is also highly prevalent in the wild boar population [20–22], suggesting that also further newly discovered atypical pestiviruses of pigs, e.g. LindaV, may be present in wild boar.

The pestivirus genome consists of a single-stranded, positive-sense RNA molecule with a length of about 11.5 to 13 kilobases (kb) and it is organized into a single open reading frame (ORF) flanked by untranslated regions (UTRs) at the 5’ and 3’ ends [23]. The ORF of the pestivirus genome encodes a polyprotein that is co- and post-translationally processed by both, cellular and viral proteases, into the mature proteins. The structural proteins are the capsid protein (C), which forms the viral core, and the three envelope glycoproteins E^rns^, E1 and E2. The non-structural proteins include N^pro^, p7, NS2-NS3 (NS2, NS3), NS4A, NS4B, NS5A and NS5B [24]. The major antigenic target for the host immune response is the E2 protein, which induces neutralizing antibody and cytotoxic T-lymphocyte responses. Further, the E2 protein forms heterodimers with E1 and these E1-E2 dimers are essential for viral entry and infectivity [25].

Within the quite diverse pestivirus group, the surface proteins can be exchanged by reverse genetics systems, enabling the creation of chimeric viruses [26, 27]. In a previous study, it was shown that the heterologous expression of the E1 and E2 proteins of a newly discovered pestivirus in the backbone of a well-known representative of this virus group can be used for serological monitoring studies as soon as the genetic information of the main immunogen is available [15]. Here, we made use of this concept to express the major antigenic protein of LindaV and to subsequently investigate wild boar samples for the presence of neutralizing antibodies against this virus.

## Materials and methods

### Diagnostic samples

A total of 200 wild boar serum or plasma samples were collected postmortem by local hunters in July (*n* = 30), August (*n* = 74), September (*n* = 85) and October 2022 (*n* = 11) in the south of the German federal state of Bavaria, which borders Austria. With a sample size of 200, a prevalence of at least 1.5% can be detected with 95% confidence (calculated by https://shiny.fli.de/ife-apps/EpiRechner/). All samples were tested by a microneutralization test against BuPV expressing the E1 and E2 proteins of LindaV (BV_E1E2_LV).

### Construction, recovery and growth characteristics of the chimeric pestivirus BV_E1E2_LV

The chimeric construct pA/BV_E1E2_LV was generated on the basis of the infectious full-length BuPV cDNA clone pA/BV [28]. In this backbone, the E1 and E2 encoding sequences were replaced by the ones of the LindaV strain Austria1 (NCBI GenBank accession number KY436034) using a modified fusion PCR [26]. The genomic region encoding E1 and E2 of LindaV was amplified by PCR from a synthetic ORF (Geneart AG, Regensburg, Germany) by using Phusion High-Fidelity DNA Polymerase (New England Biolabs, Frankfurt am Main, Germany) and the primers Ph_Bungo_E1_Linda_F (5’-AAT ATT CGG AGC TGA AGC CAC ACC ATA CTG CAA TGT GAC-3’) and Ph_Bungo_E2_Linda_R (5’-GAG CCA CTA TGT TCT CGT CTG CGC CCA CTT GGT AGT G-3’). Next, the purified amplicon was used as a megaprimer in a fusion PCR with the plasmid pA/BV as template.

**Fig. 1:**
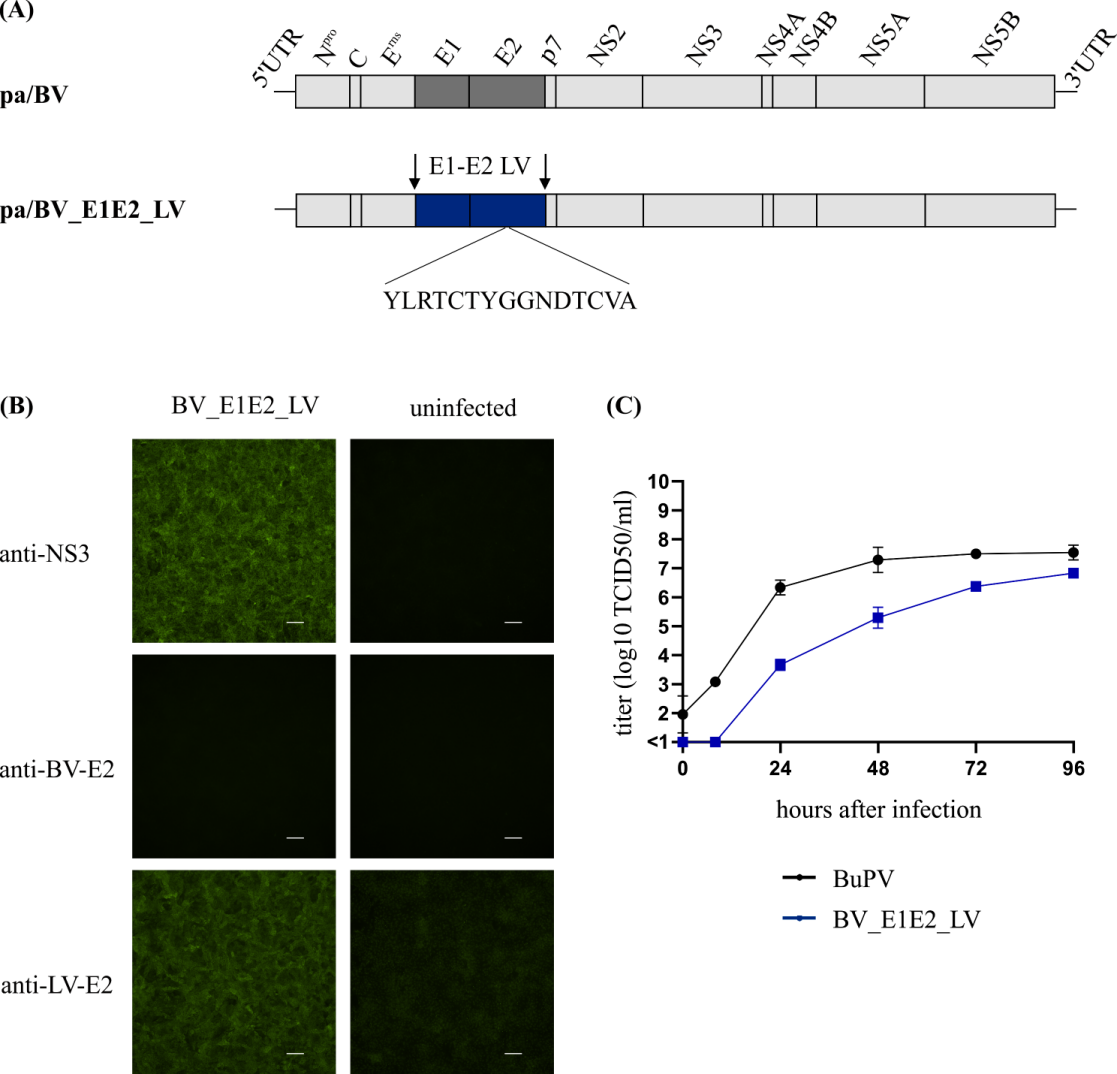
(**A**) Genome structure of the parental full-length cDNA clone pA/BV and of the newly generated chimeric construct pa/BV_E1E2_LV. The substituted structural proteins E1 and E2 of Linda virus (LindaV) are presented in blue. (**B**) Immunofluorescence staining of SK6 cells infected with Bungowannah virus (BuPV) expressing the E1 and E2 protein of LindaV (chimeric virus BV_E1E2_LV). Uninfected cells were used as controls. A pan-pesti NS3 specific antibody (anti-NS3), a BuPV specific monoclonal E2 antibody (anti-BV-E2) and a serum of a rabbit immunized with a recombinant LindaV E2 protein (anti-LV-E2) were applied. Scale bars indicate 100 μm. (**C**) Growth characteristics of the parental BuPV and of the chimeric virus BV_E1E2_LV in SK6 cells

Since this construct did not result in viable virus (data not shown) the amino acid (aa) valine at position 885 was changed to a cysteine as in the recently detected LindaV strain Austria2 (NCBI GenBank accession number MZ027894) [13] (Fig. 1). In addition to being found in the LindaV strain Austria2, a cysteine at this position is common to the classical pestivirus species such as *Pestivirus bovis* and *Pestivirus tauri* and some newly discovered atypical pestiviruses including BuPV and the Phocoena pestivirus (PhoPeV). For the aa exchange the primers Ph_Linda_MZ_mut_F (5’-TAC TTG CGC *ACG TGC ACC TAT GGG GGC A*AT GAT ACA TG-3’) and Linda_3612_R (5’-TGC GCC CAC TTG GTA GTG A-3’) were applied.

For recovery of infectious virus, the in vitro-transcribed RNA of the full-length chimeric construct was transfected into SK-6 cells (RIE262, Collection of Cell Lines in Veterinary Medicine (CCLV), Friedrich-Loeffler-Institut, Insel Riems, Germany) by using electroporation as described previously [26]. The transfected cells were grown at 37 °C and 5% CO_2_ in Minimum Essential Medium (MEM) supplemented with 10% BVDV-free fetal calf serum (FCS). Supernatants of the transfected cells were harvested two days after transfection and passaged on SK-6 cells. Virus replication was monitored by immunofluorescence (IF) staining using the pan-pesti NS3 specific antibody WB112 (APHA Scientific, Animal and Plant Health Agency, Addlestone, United Kingdom). The expression of E1 and E2 of LindaV and the absence of these proteins of BuPV were confirmed by IF staining using the BuPV specific monoclonal E2 antibody 682/45F12 (M. Dauber, Friedrich-Loeffler-Institut, Insel Riems, Germany) and a serum of a rabbit immunized with a recombinant LindaV E2 protein.

To compare the growth characteristics of chimeric and parental virus, SK-6 cells were infected with BV_E1E2_LV and recombinant BuPV recovered from pA/BV. The third passage on SK6 cells of the respective virus was used and cells were infected with a multiplicity of infection (MOI) of 0.1. Cell culture supernatants were collected at 0, 24, 48, 72 and 96 h post infection (p.i.), titrated on SK-6 cells, and virus titers were calculated as a 50% tissue culture infective dose per ml (TCID_50_/ml) after IF staining using the pan-pesti NS3 specific antibody WB112.

Virus stock for neutralization assays was prepared after three cell culture passages. The identity of the recombinant virus was confirmed by sequence analysis using appropriate primers.

### Neutralization test

The microneutralization test was performed as described for BVDV in the German official collection of test methods for bovine viral diarrhea [29]. Briefly, two-fold dilutions of the sera were prepared in MEM (starting dilution 1/5) and 50 µl of the diluted sera were incubated with 50 µl of MEM containing 100 TCID_50_ of BV_E1E2_LV in microtiter plates for 2 h at 37 °C. Thereafter, 100 µl of a suspension of SK-6 cells in MEM containing 10% FCS was added and the microtiter plates were incubated for 3 days at 37 °C. The cells were subsequently fixed by heat (80 °C, 2 h). Evaluation was done by IF staining using the pan-pesti NS3 antibody WB112 and an Alexa Fluor 448-labelled anti-mouse antibody (dilution 1/1000). All sera were tested in three replicates and the antibody titer was calculated as ND_50_ according to Behrens and Kaerber. As positive control, the serum of a rabbit immunized with the E2 protein of LindaV was included.

The recombinant E2 protein used for immunization of the rabbit was expressed in Expi293 cells. Therefore, the E2 glycoprotein of LindaV (aa 703–1048 of the polyprotein, NCBI GenBank accession number KY436034) was cloned in the expression vector pEXPR103 (iba lifesciences, Göttingen, Germany) using a codon-optimized DNA string fragment as a template (GeneArt, Thermo Scientific, Darmstadt, Germany). The plasmid DNA was transfected using the ExpiFectamine293 transfection kit (Thermo Scientific) according to the manufacturer´s instructions. The supernatant was harvested 5 days after transfection and the protein was purified using Strep-Tactin XT Superflow high capacity resin (iba lifesciences) according to the protocol of the manufacturer. A rabbit was immunized four times at 4-week intervals each with 50 µg of protein supplemented with 10% (v/v) POLYGEN (permission number LALLF 7221.3-2-042/17, State Office for Agriculture, Food Safety and Fisheries of Mecklenburg-Vorpommern, Rostock, Germany). The serum sample that was used as a positive control for the microneutralization test was taken two weeks after the final immunization.

## Results and discussion

As the major surface protein of pestiviruses, the E2 glycoprotein generally induces a strong serological response after infection [30–32]. Furthermore, it was repeatedly shown that the surface glycoproteins are interchangeable by reverse genetics methods, enabling the expression of the E2 of a pestivirus by another representative of this virus group [15, 26, 27, 33, 34]. In more distantly related pestiviruses, a combined substitution of both the E1 and the E2 protein may be necessary for the rescue of a recombinant chimeric virus [26], and this approach was also selected in the present study. E1 by itself does not induce a significant antibody response, but is incorporated into the viral envelope in a heterodimeric complex with E2 [35] and, therefore, a substitution of both glycoproteins could be beneficial for the generation of infectious virus in a chimeric setup.

Here, chimeric pestivirus BV_E1E2_LV could be recovered after RNA transfection and was efficiently passaged on SK-6 cells. The expression of LindaV-E1 and -E2 and the absence of BuPV-E1 and -E2, respectively, were confirmed by IF staining (Fig. 1). Although BV_E1E2_LV growth started slightly delayed compared to the parental recombinant BuPV, the differences in the course of the growth kinetics were only marginal and both viruses reached similar infectious titers at 96 h p.i. (Fig. 1), proving once more the interchangeability of the surface proteins between different related members of the genus *Pestivirus*. It also shows, that both BuPV and LindaPV envelope proteins have very similar functions and can substitute for each other.

When tested in the neutralization assay against the chimeric virus BV_E1E2_LV, the rabbit serum raised against E2 of LindaV gave a very high titer (1/40,960), thereby confirming the functionality of the test system. In contrast to the positive control serum, all wild boar samples tested negative (titers < 1/5).

Hence, there is no indication that LindaV spread in the investigated wild boar population.

So far, LindaV or specific antibodies have only been found in domestic pigs in Austria [11, 13, 14], but epidemiological studies in other animal species, such as wild boar, are not yet available. Therefore, the true host range is largely unknown. For other swine-infecting pestiviruses or further porcine viruses, wild boar populations have been identified as a significant reservoir [18–22, 36]. Therefore, we serologically investigated wild boar living in a region that borders the country in which LindaV has been detected recently. The absence of neutralizing antibodies in the analyzed sample pool indicates that LindaV is not present at higher prevalence (> 1.5%) in the monitored population. However, experimental infection studies under well-defined conditions are needed to exclude wild boar as a possible host. In addition, testing of wildlife in contact or close proximity to acutely affected commercial pig farms might be beneficial to define the role of wild boar in the epidemiology of the virus. Nevertheless, our results demonstrate that LindaV did not become highly prevalent in the central European wild boar population, as it has been the case for APPeV, another recently discovered atypical pestivirus, which was initially described in 2015 [8] and subsequently detected worldwide in both, domestic pigs and wild boar [9, 20–22].

## Data Availability

All data generated or analyzed during this study are included in this published article.

## Abbreviations

aa: Amino acid
APPeV: Atypical porcine pestivirus
BDV: Border disease virus
BuPV: Bungowannah virus
BVDV: Bovine viral diarrhea virus
C: Capsid protein
CCLV: Collection of Cell Lines in Veterinary Medicine
CSFV: Classical swine fever virus
CT: Congenital tremor
FCS: Fetal calf serum
IF: Immunofluorescence
LindaV: Lateral-shaking inducing neuro-degenerative agent virus
MEM: Minimum Essential Medium
ORF: Open reading frame
p.i.: Post infection
UTR: Untranslated region
